# Principles of perceptual grouping: implications for image-guided surgery

**DOI:** 10.3389/fpsyg.2015.01565

**Published:** 2015-10-20

**Authors:** Birgitta Dresp-Langley

**Affiliations:** ICube UMR 7357 Centre National de la Recherche Scientifique, University of StrasbourgStrasbourg, France

**Keywords:** *Gestalt* theory, law of *good continuation*, principle of *Prägnanz*, collinear integration, border ownership, figure-ground, image-guided surgery

The laws and principles which predict how perceptual qualities can be extracted from the most elementary visual signals were discovered by the *Gestalt* psychologists (e.g., Wertheimer, 1923; Metzger, 1930, translated and re-edited by Spillmann in 2009 and 2012, respectively). Their seminal work has inspired visual science ever since, and has led to exciting discoveries which have confirmed the *Gestalt* idea that the human brain would have an astonishing capacity for selecting and combining critical visual signals to generate output representations for decision making and action. This capacity of selection and integration enables the perception of form and space, and the correct estimation of relative positions, trajectories, and distances of objects represented in planar images. The *Gestalt* laws and principles were initially aimed at answering a single all-encompassing question: “Why does the world look the way it does.” They have subsequently been made operational in experimental studies (for an illustration of on-going research see the international METHUSALEM project, coordinated by Johan Wagemans, at www.gestaltrevision.be) aimed at deepening our insights into the ways in which specific characteristics and qualities of visual configurations may determine perceptual organization and behavior at various levels of processing. Perceptual organization directly determines the ability of human observers to assess (1) which parts of an image belong together to form a unified visual object or shape, and (2) which parts should be nearer and which further away from the observer if the represented objects were seen in the real world. This opinion paper argues that the Gestalt principle of *Prägnanz* and the Gestalt law of *good continuation* address specific problems of perceptual organization with critical implications for visual interface design, and the design of image-guided surgery platforms in particular.

The principle of *Prägnanz* relates to the general *Gestalt* postulate that objects in the visual field will produce the simplest and most complete perceptual solution possible under the conditions given. The *Gestalt* laws of perceptual organization, of which the law of *good continuation* is a particular example, describe the conditions under which specific perceptual solutions (groupings) are likely to occur. The question of how planar image structures are grouped into perceptual representations of figure and ground is one of the study grounds the *Gestalt* laws have been designed for. Figure-ground representation is a perceptual solution that enables the observer to assess which objects in the image would be likely to be nearer and which objects would be likely to be further away in a real world configuration. It is mediated by specific image cues to shape and to relative distance, involving local signals of contrast and orientation to fill in specific regions of an image and thereby enabling the perception of surfaces. The associated perceptual sensations of local contrast enhancement make visual objects in the image appear to stand in front of other objects represented in the same plane. Such sensations are often deemed “illusory” because they have no physical origin, i.e., there is no objective difference in local luminance that would explain the resulting percepts (e.g., Heinemann, 1955; Hamada, 1985; O'shea et al., 1994; De Weert and Spillmann, 1995; Grossberg, 1997; Dresp and Fischer, 2001; Dresp et al., 2002; Guibal and Dresp, 2004; Devinck et al., 2006; Pinna and Reeves, 2006; Dresp-Langley and Reeves, 2012, 2014). An essential aspect of this process of figure-ground segregation is the perceptual assignment of border ownership (see the review by von der Heydt on this topic). The *Gestalt* theorist Rubin (1921) was among the first to point out that a figure has distinct perceptual qualities that make it stand out against the rest of the visual field, which thereby acquires the perceptual quality of ground (or background). A figure occludes the ground and, therefore, owns the borders which separate it from the latter (Craft et al., 2007; Zhang and von der Heydt, 2010). Zhou et al. (2000) found neurons predominantly in V2 (but also V1) of the monkey that respond selectively to the location of borders in the visual field. Selective visual attention to the figure strengthens the neuronal responses to its borders (Qiu et al., 2007).

The *Gestalt* psychologists also correctly presumed that, to recover a representation of a whole from parts, the brain must achieve the perceptual integration of visual information across collinear space (e.g., Wertheimer, 1923; Metzger, 1930). The visual integration of contrast information across collinear image space plays a crucial role in form vision under conditions of stimulus uncertainty and configurative ambiguity (e.g., Dresp, 1997; Grossberg, 1997). It is governed by the so-called *law of good continuation*, and reflected by interactive effects between co-axial stimuli in the visual field (Hubel and Wiesel, 1959, 1968; von der Heydt and Peterhans, 1989; Dresp and Bonnet, 1991; Peterhans von der Heydt, 1991; Kapadia et al., 2000; Craft et al., 2007). Specific response activities of visual cortical neurons are triggered by these co-axial interactions (cf. the first observations by Nelson and Frost, 1978; von der Heydt et al., 1984 in monkey visual cortex), revealing the functional properties of brain mechanisms designed to complete physically discontinuous contrast input across collinear visual space. Collinear spatial integration is crucial for the detection of alignment, virtual trajectories, and shape borders in a world where most objects are seen incompletely. It enables a human observer to assess the continuity of image fragments under conditions of diminished visibility and heightened stimulus ambiguity. Experimental data on collinear visual integration have shown that the perceptual recovery of global representations of collinear space involves many levels of visual processing, not a single one, from the visual detection of local image detail to the perception of global association fields (e.g., Dresp, 1993; Field et al., 1993; Polat and Sagi, 1993, 1994; Kapadia et al., 1995; Polat and Norcia, 1996; Yu and Levi, 1997, 2000; Wehrhahn and Dresp, 1998; Chen et al., 2001; Chen and Tyler, 2001; Tzvetanov and Dresp, 2002; Dresp and Langley, 2005; Chen and Tyler, 2008; Huang et al., 2012). In complex images, some visible stimulus fragments appear clearly aligned, others do not. Specific phenomenal conditions of contour relatability (Kellman and Shipley, 1991; Shipley and Kellman, 1992, 2001) need to be satisfied to enable collinear interpolation in static 2D scenes. This process of interpolation constrains the spreading of surfaces across unspecified regions in the image. The contribution of past experience and perceptual learning to early mechanisms of interpolation and grouping needs to be taken into account given that specific memory data about objects (Kimchi and Hadad, 2002) and their most likely spatial configuration are likely to facilitate (or eventually interfere with, depending on conditions) ongoing visual processing of an image.

Although the recovery of veridical object properties was not a major question in early Gestalt theory, its laws of perceptual organization have generated a conceptual framework for addressing it. Understanding which image conditions produce geometric configurations that will satisfy the most essential laws of *Gestalt* and ensure optimal *Prägnanz* for image based decision making is similar to understanding the grammar of well-formed sentences. Gestalt theory is as relevant as ever in the context of visual interface technology for image-guided surgery, for example. Image-guided surgery uses images taken before and/or during the procedure to help the surgeon navigate. The goal is to augment the surgeon's capacity for decision making and action during the procedure (see Perrin et al., 2009, for review). In augmented reality, the guidance is provided directly on the surgeon's view of the patient by mixing real and virtual images (Figure 1). The perceptual qualities (color, brightness, salience e.a.) of the rendered images are essential for making specific regions of interest to the surgeon optimally perceptible. This includes the visual traceability of devices relative to the patient, the registration and alignment of the preoperative model, and optimized rendering and visualization of the preoperative data. Visualization in this context means translating image data into a graphic representation that is understandable by the user (the surgeon), as it conveys important information for assessing structure and function, and for making (the right!) decisions during an intervention. The field has evolved dramatically in recent years, yet, the most critical problem for image-guided surgery is still the one of task-centered user interface design. During a surgical intervention, the timing of the generation of image data is absolutely critical, and to facilitate navigation through large cavities with multiple potential obstacles, such as within the abdomen, complex displays have been designed to provide navigational aids. They combine surface renderings of anatomy (Figure 1, middle) from preoperative imaging with intra-operative visualization techniques. A common strategy here is representing volumetric data as 2D surfaces with varying opacity. The efficiency of renderings for facilitating decisions of the human user can be evaluated in terms of the perceptual salience of critical surfaces that represent regions of interest to the surgeon.

**Figure 1:**
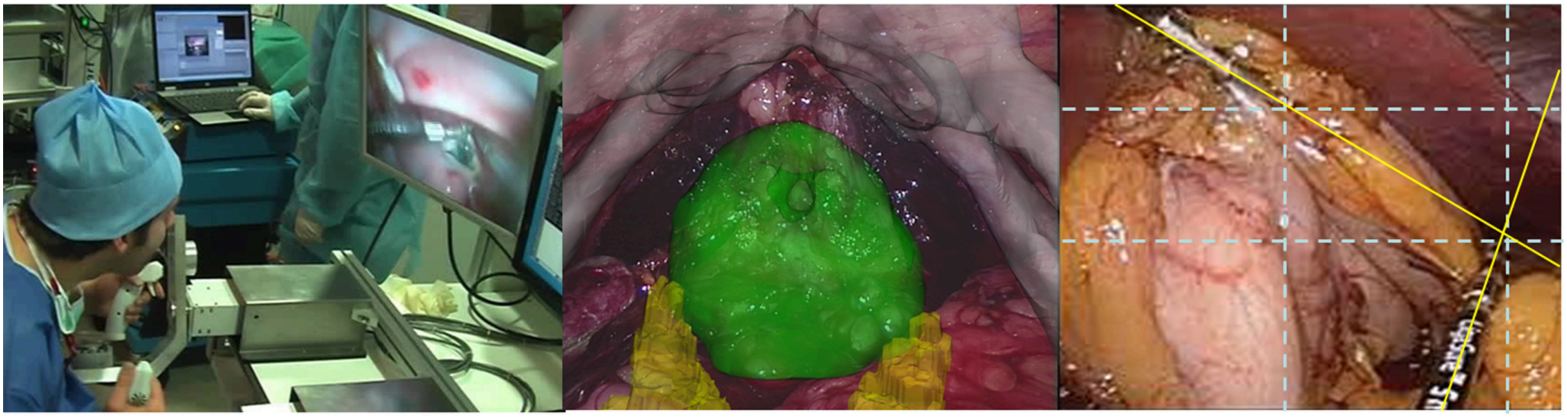
**An image configuration will produce the simplest and most complete perceptual solution possible under the conditions given (***Gestalt*** principle of ***Prägnanz***)**. In image-guided surgery, visual guidance is provided directly on the surgeon's view of the patient's anatomy by mixing real and virtual images. Understanding which image conditions produce geometric configurations that will satisfy the most essential laws of *Gestalt* and ensure optimal *Prägnanz* for decision will help increase the efficiency of rendered images **(middle)**. The goal here is to facilitate interventional strategies with regard to specific regions of interest to the surgeon. Visual tracking of the tooltip trajectories is important for evaluating skill evolution, the positional accuracy of the tooltips being critical **(left)**. Technology facilitating the positional accuracy of tool-tip movements by generating visual data for relative position, alignment, and trajectory anticipation (perceptual law of *good continuation*) is urgently needed. The real-time computational analysis of deviations from critical alignments during interventions **(right)** is currently the “holy grail” in this field of technological development.

Moreover, intra-operative imaging often provides further diagnostic information and permits assessing risks as well as perspectives of repair. In this context, image-guided instrument tracking is a major challenge for current research and development in this field (West and Maurer, 2004; Huang et al., 2007). A critical problem for the surgeon is detecting and keeping track of the relative positions of the surgical tools he/she is using during the intervention (Figure 1, right). Visual tracking of the tooltip trajectories is also a precious aid for evaluating skill evolution in trainee surgeons, the positional accuracy of the tooltips being critical during an intervention (e.g., Jiang et al., 2015). The development and testing of new visual aids to facilitate the detection of alignment, relative position and trajectories (perceptual law of *good continuation*) is urgently needed here. Ultimately, technology where the surgical tool itself will become a genuine visual navigation aid in image-guided surgery is to be developed in the near future and psychophysical testing should have a major impact on these developments.

## Funding

Grant support was provided by the Centre National de la Recherche Scientifique (CNRS MI AAP 2015).

### Conflict of interest statement

The author declares that the research was conducted in the absence of any commercial or financial relationships that could be construed as a potential conflict of interest.
